# Supplementary material for: Effect of synchronization of firings of different motor unit types on the force variability in a model of the rat medial gastrocnemius muscle
Source: PLoS Comput Biol. 2021 Apr 26;17(4):e1008282. doi: 10.1371/journal.pcbi.1008282 (PMC8101995; doi:10.1371/journal.pcbi.1008282)
Supplement: S1 Fig — Normalized histograms of inter pulse intervals of 57 motor units (MUs) of a rat muscle gastrocnemius model, including: 8 slow (S) MUs, 23 fast resistant to fatigue (FR) MUs, and 26 fast fatigable (FF) MUs. (DOCX) [file pcbi.1008282.s003.docx]

**Figure 1Sup**

**supplementary material**

**Normalized histograms of inter-pulse intervals in 57 Motor Units (MUs) of a rat muscle gastrocnemius model, including:**

**8 slow (S) MUs, 23 fast resistant to fatigue (FR) MUs, and 26 fast fatigable (FF) MUs.**

**Basic (non-synchronized) model**

**MU synchronization by Method 1 (**Δ**t** ± **6 ms)**

**MU synchronization by Method 2 (**Δ**t** ± **6 ms)**

**MU synchronization by Method 3 (**Δ**t** ± **6 ms)**

**MU synchronization by Method 4 (**Δ**t** ± **6 ms)**
